# Targeted gene deletion with *Sp*Cas9 and multiple guide RNAs in *Arabidopsis thaliana*: four are better than two

**DOI:** 10.1186/s13007-023-01010-4

**Published:** 2023-03-28

**Authors:** Jana Ordon, Niklas Kiel, Dieter Becker, Carola Kretschmer, Paul Schulze-Lefert, Johannes Stuttmann

**Affiliations:** 1 Department of Plant-Microbe Interactions, Max-Planck Institute for Plant Breeding Research, D50829 Cologne, Germany; 2 Cluster of Excellence on Plant Sciences (CEPLAS), Max Planck Institute for Plant Breeding Research, Cologne, Germany; 3 Institute for Biology, Department of Plant Genetics, Martin Luther University Halle-Wittenberg, D06120 Halle, Germany; 4 Institute for Biosafety in Plant Biotechnology, Federal Research Centre for Cultivated Plants, Julius Kühn-Institute (JKI), 06484 Quedlinburg, Germany; 5 CEA, CNRS, BIAM, UMR7265, LEMiRE (Rhizosphère et Interactions sol-plante-microbiote), Aix Marseille University, 13115 Saint-Paul lez Durance, France

**Keywords:** CRISPR/Cas, *Sp*Cas9, Chromosomal deletion, Gene deletion, Arabidopsis, TREX2

## Abstract

**Background:**

In plant genome editing, RNA-guided nucleases such as Cas9 from *Streptococcus pyogenes* (*Sp*Cas9) predominantly induce small insertions or deletions at target sites. This can be used for inactivation of protein-coding genes by frame shift mutations. However, in some cases, it may be advantageous to delete larger chromosomal segments. This is achieved by simultaneously inducing double strand breaks upstream and downstream of the segment to be deleted. Experimental approaches for the deletion of larger chromosomal segments have not been systematically evaluated.

**Results:**

We designed three pairs of guide RNAs for deletion of a ~ 2.2 kb chromosomal segment containing the Arabidopsis *WRKY30* locus. We tested how the combination of guide RNA pairs and co-expression of the exonuclease TREX2 affect the frequency of *wrky30* deletions in editing experiments. Our data demonstrate that compared to one pair of guide RNAs, two pairs increase the frequency of chromosomal deletions. The exonuclease TREX2 enhanced mutation frequency at individual target sites and shifted the mutation profile towards larger deletions. However, TREX2 did not elevate the frequency of chromosomal segment deletions.

**Conclusions:**

Multiplex editing with at least two pairs of guide RNAs (four guide RNAs in total) elevates the frequency of chromosomal segment deletions at least at the *AtWRKY30* locus, and thus simplifies the selection of corresponding mutants. Co-expression of the TREX2 exonuclease can be used as a general strategy to increase editing efficiency in Arabidopsis without obvious negative effects.

**Supplementary Information:**

The online version contains supplementary material available at 10.1186/s13007-023-01010-4.

## Background

Since the discovery of the mode of action of RNA-guided nucleases (RGNs; [10, 19, 25, 33], Cas9 from *Streptococcus pyogenes* (*Sp*Cas9) has become a routine tool for genome editing in many plant species. For mutagenesis of protein-coding genes, it is generally sufficient to program Cas9 for cleavage at a single target site within the gene of interest. Resulting double-strand breaks (DSBs) are mainly repaired by non-homologous end joining (NHEJ) in plant cells. As an error-prone process involving repeated RGN-mediated DNA cleavage upon precise repair, NHEJ frequently provokes small insertions or deletions at the initial DSB site. Indeed, + 1/–1 nucleotide insertions/deletions (InDels) are the most frequently detected polymorphisms in CRISPR mutagenesis [6, 29]. These small InDels provoke frame-shift mutations, which result in the disruption of protein-coding genes.

However, in a number of scenarios, it may be preferable to induce the deletion of a chromosomal segment. This may be the case, *e.g.*, during mutagenesis of promoter regions to alter gene expression, functional interrogation of other non-coding sequences or deletion of gene clusters [15, 22, 36, 39, 47]. Also, remaining gene fragments may retain functionality, or the presence of alternative start codons [2] downstream of a target site or alternative splicing may lead to expression of a functional mRNA even after the introduction of small InDels. This can be prevented or excluded by the deletion of the full coding sequence. Further, chromosomal segment deletions can be induced to determine whether a gene has essential functions.

In site-specific mutagenesis with *Sp*Cas9 or other RGNs, bi-allelic mutations are often induced directly in primary transformants [22, 49]. Thus, if an essential gene is targeted within the coding sequence, the majority of primary transformants will not survive. Obtaining informative material from such editing approaches requires discovery/isolation of primary transformants that are heterozygous for deleterious mutations, or that carry hypomorphic alleles. These two events are rare and cannot be specifically selected. *Sp*Cas9-induced chromosomal segment deletions often occur as hemizygous events in primary transformants (*e.g.*, [40]. The second chromosomal copy may or not carry small InDels at individual *Sp*Cas9 target sites. Thus, if a chromosomal segment deletion encompasses an essential gene (but small InDels at individual target sites do not affect gene function), hemizygous and viable primary transformants can be selected and further analyzed in a subsequent segregating population.

Chromosomal segment deletions (in the following, chromosomal deletions) are generated by inducing DSBs upstream and downstream of the targeted segment, and its loss during repair by the NHEJ mechanism. In Arabidopsis (*Arabidopsis thaliana*), we have previously observed that frequencies of chromosomal deletions decreased with deletion size, and that InDels at individual target sites were more frequent than chromosomal deletions (loss of the internal fragment; [40]). Nonetheless, Arabidopsis lines carrying large chromosomal deletions (*e.g.*, > 40–80 kb) can be conveniently isolated from screening primary transformants by PCR, especially when using highly efficient nuclease systems [22]. Also, in rice, chromosomal deletions occurred only in some transformants [54], and InDels at single target sites are more common [41]. In contrast, in tomato, chromosomal deletions were more common than mutations at individual target sites in at least one case, although a relatively small deletion (< 50 nt) was induced [35].

A pair of guide RNAs programming Cas9 for cleavage at one target site upstream and one downstream of a given chromosomal segment is sufficient for the induction of chromosomal deletions (dual targeting). However, addressing multiple up- and downstream target sites might increase the probability of losing the internal fragment and thus inducing the desired chromosomal deletion. We therefore used two pairs of guide RNAs (four guide RNAs) when we intended to induce chromosomal deletions in previous studies [22, 38–40], while others relied on dual targeting (*e.g.* [52]). A systematic comparison to deduce design guidelines for chromosomal deletion induction has not yet been conducted.

We compared here different constructs to evaluate whether increasing the number of guide RNA pairs or co-expression of a DNA exonuclease, TREX2, could enhance chromosomal deletion frequencies in Arabidopsis. We chose the *WRKY30* locus for deletion. We show that increasing the number of guide RNAs from two to four enhanced frequencies of chromosomal deletions encompassing the *WRKY30* locus. In fact, we could detect bi-allelic chromosomal deletions among primary transformants only when we edited with four guide RNAs. This facilitated the isolation of transgene-free *wrky30* mutants in the T_2_ generation without further screening. We confirmed mutant lines by long-read (PacBio) sequencing in the T_3_ generation. Co-expression of TREX2 exonuclease did not enhance the frequency of chromosomal deletions. However, TREX2 increased the frequency of InDel mutations at individual target sites two-fold and shifted the mutation spectrum towards larger deletions without adverse effects. Thus, co-expression of TREX2 can be used to augment mutation frequency during site-specific mutagenesis.

## Results

### Editing of the *WRKY30* locus in *Arabidopsis thaliana*

We aimed to generate an Arabidopsis *wrky30* mutant line by gene deletion (chromosomal deletion), as we assumed *WRKY30* might be an essential gene [32, 43, 55]. Using this locus as a case study, we investigated whether increasing the number of guide RNA pairs and/or co-expression of the exonuclease TREX2 can enhance the frequency of chromosomal deletions.

**Fig. 1:**
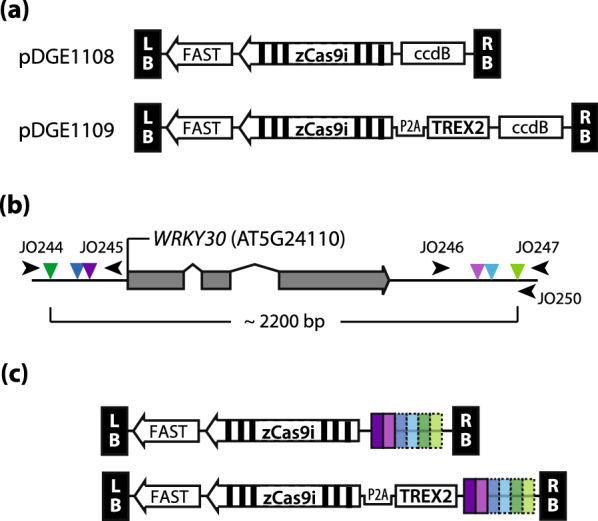
Constructs and target selection for deletion of the *AtWRKY30* locus. **a** Recipient vectors pDGE1108 and pDGE1109. The FAST (Fluorescence Accumulating Seed Technology) marker allows positive and negative selection of transgenics. The intron-optimized *zCas9i* gene is under control of the Arabidopsis *RPS5a* (*Ribosomal Particle S5a*) promoter and a chimeric terminator (“t-triple “: t35S-tNbACT-Rb7MAR; [12]). **b** Scheme of the *WRKY30* locus and selected target sites (guide RNAs). Three pairs of target sites (triangles; inner—pink/light pink, middle—blue/light blue, outer—green/light green) were selected and corresponding guide RNAs designed. Arrowheads represent binding sites of oligonucleotides used for PCR genotyping. **c** Scheme of multiplex editing vectors assembled for *WRKY30* editing and containing or not *TREX2*. Constructs contain cassettes for expression of pairs of guide RNAs, pairs of pairs, or all six guide RNAs

First, we assembled two new recipient vectors compatible with our Dicot Genome Editing (pDGE) vector toolbox, pDGE1108 and pDGE1109 (Fig. 1; [40, 45]). These vectors contain a cassette for positive/negative selection by seed fluorescence (Fluorescence Accumulating Seed Technology (FAST); [44]), the *zCas9i* gene under control of the *RPS5a* promoter [38, 46] and a “triple terminator” (t35S + tNbACT + Rb7-MAR; [12]), and a *ccdB* cassette. The *zCas9i* gene contains 13 introns [22]. We previously demonstrated that expression from the *zCas9i* gene strongly enhances Cas9 activity in Arabidopsis and other plant species [22, 45]. The *ccdB* cassette in pDGE1108/1109 is flanked by recognition sites for the Type IIs endonuclease *Bsa*I/*Eco31*I and can be replaced by one or multiple guide RNA transcriptional units by GoldenGate cloning to obtain final editing constructs (Fig. 1a, [16, 40]).

The chimeric “triple terminator” consists of the commonly used 35S terminator from Cauliflower Mosaic Virus fused to a terminator region from *Nicotiana benthamiana Actin3* and the Rb7 matrix attachment region from tobacco. In comparison to a construct containing the *nopaline synthase* (*nos*) terminator, the triple terminator could previously increase the expression of GFP by up to 60-fold [12]. We analyzed Cas9 accumulation (35S promoter control) by agroinfiltration in *N. benthamiana* leaves. In comparison to the rbcS-E9 terminator [38, 49], expression of Cas9 terminated by the chimeric triple terminator or by a tobacco extensin terminator (tNbEU; [12]) resulted in a mild increase in protein accumulation (Additional file 1: Fig S1).

In pDGE1109, the Cas9 expression cassette furthermore contains the *TREX2* coding sequence, fused 5’ to *zCas9i*, and separated by a P2A peptide-coding sequence (Fig. 1a). When combined with a sequence-specific nuclease, exonucleases such as TREX2 promote end resection and thus augment mutagenesis frequency by increasing the error rate during NHEJ [3, 4]. P2A mediates ribosomal skipping and thus the synthesis of TREX2 and Cas9 as individual polypeptides from a single mRNA [13, 48]. We expressed *TREX2(P2A)-zCas9i* in agroinfiltration experiments conducted in two different laboratories. In one condition, a single band comparable in intensity to that of Cas9 (without TREX2) was detected on immunoblots using a Cas9-specific antibody (Additional file 1: Fig S1b). In the other condition, an additional higher molecular weight signal most likely corresponding to a TREX2-Cas9 translational fusion product was detected (Additional file 1: Fig S1c). We conclude that read-through may be detectable for the used P2A sequence at least in some cases.

To induce chromosomal deletions encompassing the *WRKY30* locus, we selected three pairs of target sites in flanking sequences (Fig. 1b). The minimal/maximal distances between Cas9 cleavage sites were ~ 1830 and 2160 bp, respectively. The average gene size in Arabidopsis is approximately 2200 bp [11]. The chosen deletion size is thus representative and applicable for deletion of many Arabidopsis genes. We designed guide RNAs corresponding to the selected target sites, and assembled constructs for expression of guide RNA pairs, pairs of guide RNA pairs, or all six guide RNAs, in both pDGE1108 and pDGE1109 (Fig. 1c). This resulted in 14 different constructs (Table 1), which were transformed into Arabidopsis accession Col-0 by floral dipping.

**Table 1:**
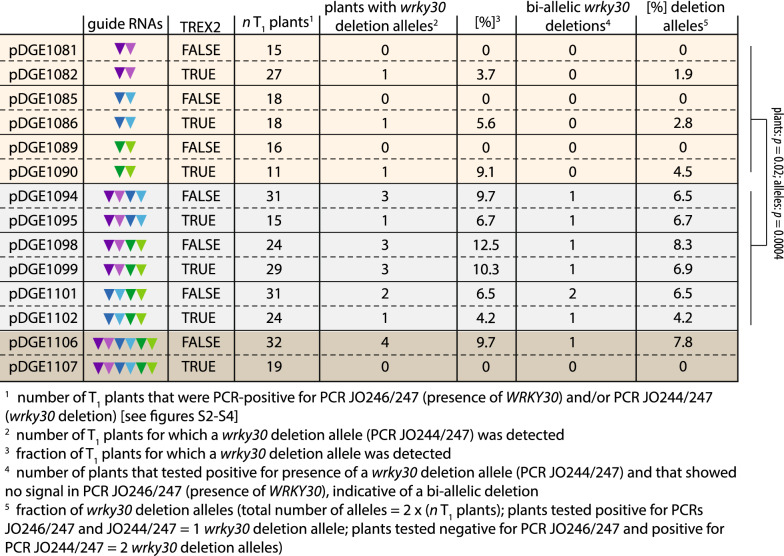
Chromosmal deletion frequency in primary Arabidopsis transformants

### Multiple cuts into flanking DNA genomic sequences increase chromosomal deletion frequency

We assessed the frequency of chromosomal deletions at the *WRKY30* locus by PCR screening in the T_1_ generation (Table 1, Additional file 2: Fig S2, Additional file 3: Fig S3, Additional file 4: Fig S4). Primary transformants were selected by seed fluorescence, and T_1_ plants (n = 11–32) genotyped using primers flanking the *WRKY30* locus and *Sp*Cas9 target sites (Fig. 1b; JO244/247). Among transformants harboring constructs with guide RNA pairs (two guide RNAs), chromosomal deletions were detected at low frequencies. Bi-allelic *wrky30* deletions were not detected. Importantly, the frequency of *wrky30* deletion alleles increased when pairs of guide RNA pairs (4 guide RNAs) were expressed (Table 1). In this case, candidate bi-allelic *wrky30* deletion lines were recovered from all T_1_ populations. The frequency of chromosomal deletions was significantly elevated at the level of plants with *wrky30* deletion alleles (Student`s t-test, p = 0.02) and when comparing the absolute number of chromosomal deletions (p = 0.0004) between constructs with two or four guide RNAs (Table 1). When editing with six guide RNAs, chromosomal deletions encompassing the *WRKY30* locus were detected in transformants expressing Cas9 (without TREX2) at frequencies similar to those obtained when editing with four guide RNAs. No chromosomal deletions were detected in transformants expressing six guide RNAs and TREX2-zCas9i (Table 1, Additional file 4: Fig S4). No significant differences were detected when comparing plants with chromosomal deletions or the absolute number of chromosomal deletions between populations with or without *TREX2* (Student`s t-test; p = 0.93 or p = 0.87, respectively).

Overall, we conclude that using four guide RNAs instead of two increases the frequency of chromosomal deletions. Expression of six guide RNAs did not further elevate chromosomal deletion frequency in our experiments; a result based on a limited number of observations. We did not detect a difference in chromosomal deletion frequency with simultaneous expression of TREX2 and Cas9.

### Co-expression of the exonuclease TREX2 elevates mutation frequency and results in larger deletions

**Fig. 2:**
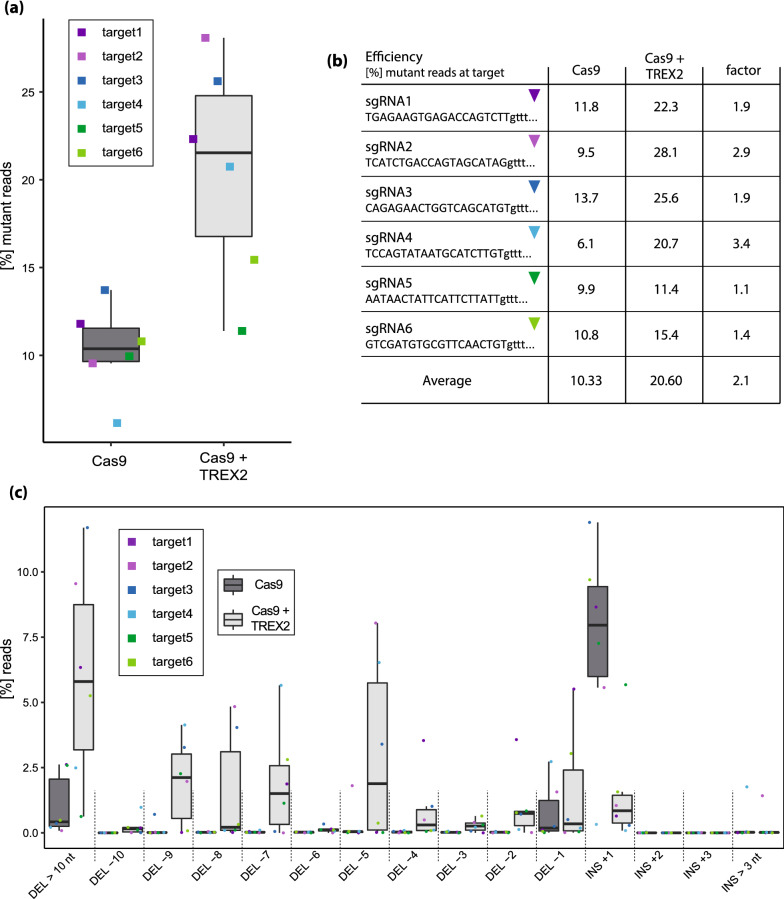
TREX2 improves editing efficiency and alters mutation profiles toward larger deletions. **a** Overall mutation frequencies at the six target sites with constructs containing or not *TREX2*. Amplicons containing the target sites up- or downstream of *WRKY30* were PCR-amplified using DNA from pooled T_1_ individuals (transformation of pDGE1081-1090). Up- and downstream amplicons for each construct were pooled and Illumina-sequenced. Mutation frequencies were determined using CRISPResso. Color code (target sites/sgRNAs) as in Fig. 1b. **b** Guide RNA efficiencies at single target sites. As in (**a**), but individual target sites ± *TREX2* are shown. **c** Mutation profiles over six target sites upon editing with constructs containing or not the *TREX2* exonuclease gene. Samples and data analyses as in (**a**), but frequencies of different InDels are illustrated. See also Additional file 5: Figure S5 for individual target sites

We further analyzed the effect of TREX2 and Cas9 co-expression at the level of individual target sites. We designed amplicons covering target sites up- and downstream of *WRKY30* (oligonucleotide combinations JO244/245 and JO246/247; Fig. 1b). The respective amplicons were generated using DNA of pooled T_1_ individuals from transformation of pDGE1081-1090 (coding guide RNA pairs; see Table 1 for the number of T_1_ individuals included in each pool) by PCR, and subjected to amplicon deep sequencing. On average, approximately 10% (6.1–13.7%) of reads contained mutations at target sites when only Cas9 was expressed (Fig. 2a, b). Mutation frequency was significantly elevated by TREX2 co-expression (p = 0.004, Student’s t-test) and increased on average two-fold (Fig. 2a, b). Of note, although mutation frequency was improved with TREX2 in all comparisons, the effect size was variable and did not appear to correlate with the initial efficiency of a given guide RNA.

When editing with plain Cas9, insertions of a single nucleotide were detected most frequently (Fig. 2c). Upon TREX2 co-expression, the frequency of insertions was significantly reduced and mutation profiles were shifted towards larger deletions at individual target sites (Fig. 2c), as previously reported [3, 50]. Approximately 27% of all InDel alleles were deletions of more than 10 nucleotides. Adverse effects of TREX2 co-expression, such as lowered numbers or reduced viability of primary transformants, were not observed.

In summary, we observed elevated mutation frequencies and a shift towards larger deletions at all individual target sites when TREX2 was co-expressed. On average, mutation frequencies doubled at target sites. TREX2 co-expression can thus be used as a general strategy to improve genome editing efficiencies in Arabidopsis.

### In-depth analysis of *wrky30* mutant lines and confirmation by long-read DNA sequencing

We selected T_2_ populations derived from four primary transformants for isolation of lines containing bi-allelic chromosomal deletions encompassing the *WRKY30* locus. Putative bi-allelic chromosomal deletions were detected in transformants 1094.29, 1101.12 and 1101.13 (Additional file 3: Fig S3). Transformant 1098.5 was scored heterozygous for a chromosomal deletion. We selected FAST-negative seeds from populations derived from these transformants to select against the presence of the T-DNA. T_2_ plants were sampled as pools of four plants (two pools per population), and pool DNA was used for genotyping (Additional file 6: Fig S6). In accordance with results obtained with primary transformants, bi-allelic chromosomal deletion alleles were detected in the first three pools, and a chromosomal deletion segregated in population 1098.5. Thus, chromosomal deletions detected in the T_1_ generation were germline-transmitted to the T_2_ generation in all tested populations.

**Fig. 3:**
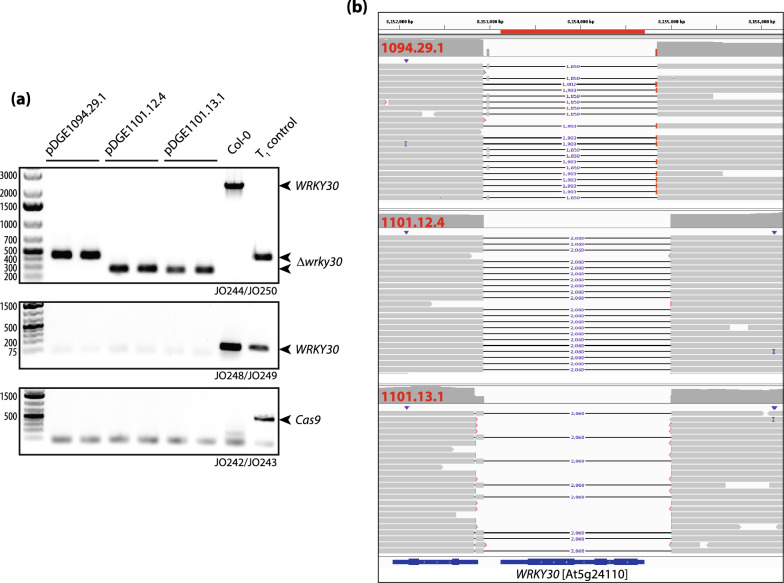
In-depth analysis of *wrky30* mutant lines by PCR and long-read DNA sequencing. **a** PCR genotyping of *wrky30* mutant lines. Pools were assembled from five randomly selected plants derived from indicated T_3_ populations; two pools per population. Corresponding DNAs were used for PCR genotyping (see Fig. 1 for primer binding sites): Amplicon JO244/250 spans the *WRKY30* locus; a smaller fragment is amplified in deletion lines. JO248/249 amplify a fragment within *WRKY30*, absent in deletion lines. JO242/243 amplify a fragment of the *zCas9i* gene to query presence/absence of the T-DNA. Col-0 and a T_1_ individual (1095.14) were included as controls. **b** Read mappings derived from long-read sequencing (Pacific Biosciences HiFi) of DNA pools (~ 20 plants) derived from indicated populations. Read mappings were visualized using Integrative Genome Viewer [42]. The *WRKY30* locus and ~ 1 kb of up-/downstream sequences is shown

Single plants were propagated to the T_3_ generation, and PCR-genotyping was repeated on pools of five plants for lines with bi-allelic chromosomal deletions (Fig. 3a). Bi-allelic deletions were confirmed, and the lack of amplification of a *zCas9i*-specific PCR product confirmed the absence of the T-DNA.

Pools of 20 plants per T_3_ population were used to extract DNA for long-read sequencing on a PacBio Sequel II system. HiFi reads were used for de novo assembly and contigs were aligned to TAIR10. Inspection of the *WRKY30* genomic region revealed that population 1094.29.1 was heterozygous for two different alleles: Chromosomal deletions of approximately 1860 bp between target sites of guide RNAs 3 and 5, and 1900 bp between guide RNAs 3 and 4 (Fig. 3b). An identical homozygous deletion of 2068 bp between guide RNAs 1 and 4 was detected in populations 1101.12.4 and 1101.13.1 (Fig. 3b). However, different mutations were detected at the target site of guide RNA6 in these lines, in agreement with independent lineages.

We used CRISPOR [9] to predict possible off-targets of guide RNAs (Additional file 8: Supplemental File S1). The respective locations were inspected in read mappings of PacBio data using IGV [42]. Mutations were not detected at any of the potential off-targeting sites.

Bi-allelic chromosomal deletions were not detected among transformants expressing two guide RNAs. We observed a higher frequency of chromosomal deletions when expressing four guide RNAs. In this case, candidate lines with bi-allelic chromosomal deletion alleles were detected in all populations tested. This enabled us to isolate clean *wrky30* deletion lines by selecting exclusively against the presence of the transgene.

## Discussion

The generation of chromosomal deletions with RGNs such as *Sp*Cas9 requires simultaneous induction of DSBs up- and downstream of a targeted chromosomal segment (Fig. 1). However, chromosomal deletions are only induced in some individuals, as InDel mutations at individual target sites are the more represented repair outcome [40, 41]. Here, we show that multiplexing with two guide RNA pairs (four guide RNAs; two target sites each up-/downstream) enhances the frequency of chromosomal deletions compared to dual targeting (two guide RNAs; one target site up-/downstream). With most of the available RGN toolkits, the additional cost of integrating four, rather than two, guide RNAs into editing constructs is insignificant. Higher frequencies of chromosomal deletions may reduce the number of plants that need to be screened for isolation of candidate mutant lines. In our case, using four guide RNAs allowed us to directly isolate candidate lines with bi-allelic chromosomal deletions in the T_1_ generation. Thus, only one selection against the transgene was required to obtain clean *wrky30* deletion lines. We conclude that four guide RNAs are better than two for inducing chromosomal deletions at least at the *AtWRKY30* locus.

RGN-induced chromosomal deletions were reported, *e.g.*, in Arabidopsis, soy bean, rice, tomato, *N. benthamiana* and *Catharanthus roseus* [14, 22, 35, 41, 54]. The frequency of chromosomal deletions in primary T_1_ transformants is strongly locus/guide RNA-dependent [40, 52]. For example, in *A. thaliana*, Wu et al. [52] report a chromosomal deletion frequency ranging from 5 to 79%, with deletions detected in 20–40% of primary transformants for most loci. In comparison, we obtained *wrky30* deletions at a moderate frequency of approximately 10% (Table 1), but obtained chromosomal deletions at higher frequencies (up to ~ 50%) at other loci with the same vector system [22],unpublished data). It should be noted that, previously, we observed inheritability of chromosomal deletions with *RPS5a* promoter-driven *zCas9i* in six of eight tested lines in the T_2_ generation [22]. In the present study, chromosomal deletions were confirmed in T_2_ in all cases. So far, chromosomal deletion alleles segregated at Mendelian ratios among non-transgenic T_2_ individuals in all populations tested. In contrast, Wu et al. [52] recovered chromosomal deletions at frequencies below 10% for most analyzed T_2_ populations (1–90%; [52]. Accordingly, corresponding primary transformants (*pcoCas9*, *Ubiquitin10* promoter control) were most likely somatic mosaics. This appears to be less of an issue with p*RPS5a*-driven *zCas9i*, or the differences might be due to tissues used for genotyping. We routinely use floral tissues for genotyping primary transformants. We assume that especially when bi-allelic chromosomal deletions are detected in floral tissues, non-inheritability or low representation of deletion alleles in the T_2_ generation is highly unlikely.

Co-expression of TREX2 with *Sp*Cas9 resulted in a two-fold increase in the efficiency of inducing InDels at individual target sites (Fig. 2). An approximately two-fold increase in mutation frequency was previously observed in tomato, barley and *Setaria viridis* protoplast experiments [3, 50]. Stable transgenic lines expressing TREX2 and Cas9, but not only Cas9, were reported for *S. viridis* [50]. Our direct comparison of efficiency at a total of six different target sites corroborates that TREX2 can robustly improve mutation frequency in stable transformants. Larger deletions (at individual target sites) obtained with TREX2 may also facilitate functional interrogation or inactivation of, *e.g.*, small non-coding RNA genes or transcription factor-binding sites [37].

TREX2 has also been reported to make genome editing at individual target sites even more precise: by avoiding repeated cleavage due to higher probability of error-prone repair, TREX2 reduces the number of translocations and large deletions that can occur, as rare events, at on-target sites [53]. Yin et al. [53] also reported that TREX2 outperformed several other tested exonucleases, and could not detect collateral damage activity. Consistent with this, we did not observe adverse effects of TREX2 co-expression in our stable Arabidopsis transgenic lines. Thus, it appears that TREX2 co-expression can be used as a general and robust strategy to elevate mutation frequency in genome editing.

We used whole genome re-sequencing with PacBio HiFi reads for verification of our mutant lines. In contrast to Sanger sequencing, this allowed us to not only determine the precise genotype at the *WRKY30* locus (Fig. 3), but also to confirm the absence of off-target mutations or, *e.g.*, translocations. At least for the moderate genome size of Arabidopsis, PacBio sequencing thus represents a cost- and labor-efficient approach for comprehensive verification of genome-edited lines.

We chose to delete the entire *WRKY30* gene rather than disrupt its coding sequence, as we supposed it could be essential [32, 43, 55]. The successful generation of bi-allelic *wrky30* deletion mutants—plants were indistinguishable from the wild type (Additional file 7: Fig S7)—demonstrates that this is not the case. However, as initially intended, multiple candidate lines hemizygous for chromosomal deletions encompassing the *WRKY30* locus were detected among primary transformants (Additional file 2: Fig S2, Additional file 3: Fig S3, Additional file 4: Fig S4, Table 1). Segregation of the *wrky30* deletion allele was confirmed for one population (1098.5.; Additional file 6: Fig S6). Accordingly, gene deletion can be used to generate material segregating for detrimental alleles in essential genes.

### Experimental outline for deletion induction with four guide RNAs in Arabidopsis thaliana

- Define chromosomal segment targeted for deletion.
- Select 200–300 bp of 5’ and 3’ flanking sequences for target site selection/guide RNA design. *E.g.*, chop-chop [28] and CRISPOR [9] are useful tools to scan for and evaluate target sites.
- Select two target sites each in 5’ and 3’ flanking sequences. Target sites should be offset to avoid steric hindrance among Cas9/guide RNA complexes. However, a large offset will lead to important differences between possible deletion outcomes, which may complicate PCR screening. We therefore recommend an offset of 50–100 bp between cleavage sites.
- Design corresponding guide RNAs, assemble construct for multiplex editing using available toolkits.
- Plant transformation. Logemann et al. [31] provided a convenient protocol for Arabidopsis transformation by floral dipping.
- Select primary transformants by FAST or antibiotic/herbicide resistance. At least 30–40 primary transformants should be obtained.
- Design PCR primers: Flanking the desired deletion, and at least one internal oligonucleotide. Screen T_1_ transformants for occurrence of deletion alleles (flanking oligonucleotides) and presence of the targeted chromosomal fragment (one flanking and one internal oligonucleotide). We preferentially use floral tissues of T_1_ transformants for DNA extraction.
- Propagate ≥ 5 plants in which a chromosomal deletion was detected to the T_2_ generation.
- Select against presence of the transgene: Non-fluorescent seeds when FAST is available.
- Repeat genotyping with T_2_ plants. Select homozygous. Confirm absence of Cas9 by PCR genotyping. Propagate selected plants to the T_3_ generation.
- Determine precise allele information by Sanger sequencing of PCR products or NGS using T_2_ or T_3_ material.

## Methods

### Plant growth conditions and transformation

*Arabidopsis thaliana* accession Columbia-0 (Col-0) plants were cultivated under short day conditions in a walk-in chamber (8 h light, 23/21 °C day/night, 60% relative humidity) or in a greenhouse under long day conditions (16 h light) for seed set. Arabidopsis was transformed by floral dipping as previously described [31]. Agrobacterium strain GV3101 pMP90 was used. Primary transformants (T_1_) were selected by seed fluorescence [44] using a stereomicroscope equipped with an mCherry filter. Plants were grown in growth chambers for genotyping, and transferred to a “speed breeding chamber” (20 h light) for seed production. In the T_2_ generation, non-fluorescent seeds were selected, respective plants genotyped and propagated to the next generation. *N. benthamiana* plants were cultivated in a greenhouse with a 16 h light period (sunlight and/or IP65 lamps [Philips] equipped with Agro 400 W bulbs [SON-T]; 130–150 μE m^−2^ s^−1^; switchpoint; 100 μE m^−2^ s^−1^), 60% relative humidity at 24/20 °C (day/night).

### Molecular cloning and guide RNA design

The GoldenGate technique following the Modular Cloning syntax for hierarchical DNA assembly was used for clonings [16, 17]. Previously reported plasmids belonging to the Modular Cloning Toolkit and the MoClo Plant Parts I and II collections were used [17, 18]. Recipient vectors pDGE1108 and pDGE1109 were assembled as previously described [40, 45]. A plasmid containing the TREX2 coding sequence was obtained via Addgene (#91026; [3]). Oligonucleotides corresponding to the target sites TGAGAAGTGAGACCAGTCTTnGG (#1), TCATCTGACCAGTAGCATAGnGG (#2), CAGAGAACTGGTCAGCATGTnGG (#3), TCCAGTATAATGCATCTTGTnGG (#4), AATAACTATTCATTCTTATTnGG (#5), and GTCGATGTGCGTTCAACTGTnGG (#6) were cloned into guide RNA shuttle vectors containing the Arabidopsis U6-26 promoter described in Stuttmann et al. [45]. Final plant transformation vectors were generated by cloning guide RNA expression cassettes into pDGE1108/1109 as previously described [45]. Target sites were selected using CRISPOR [9]. Further details are provided in Additional file 9: Supplemental File S2.

### Agroinfiltration and immunodetection

Four- to six-week-old *N. benthamiana* plants were used for agroinfiltration (OD_600_ = 0.4). Leaf discs were harvested three dpi, ground in liquid nitrogen and boiled in 2 × Laemmli buffer for protein extraction. Proteins were separated on SDS-PAA gels, and transferred to nitrocellulose or PVDF membranes by tank blotting. Monoclonal antibodies Abcam EPR18991 and Sigma-Aldrich SAB4200701 were used for detection of Cas9. HRP-conjugated secondary antibodies (GE Healthcare) were used. A mixture of SuperSignal West Pico and Femto was used for revelation on Kodak Biomax Light films or a BioRad ChemiDoc Imaging System.

### Genotyping, amplicon sequencing and data analysis

Oligonucleotides used for PCR genotyping are provided in Additional file 8: Supplemental File S1. For initial deletion screening, a DNA purification-free PCR protocol was used [24]. A standard CTAB protocol was used for further DNA extractions. For amplicon sequencing, PCR products were prepared on DNA of pooled T_1_ individuals using oligonucleotides JO244/245 and JO246/247, purified using a column kit and quantified on a Qbit. Amplicons JO244/245 and JO246/247 were pooled for each group of transformants, and sequenced by Genewiz (Amplicon-EZ). Between 42 and 62 k reads were obtained for each amplicon pool. Data were analyzed using CRISPResso2 [8]. Between 19 and 32 k reads were aligned to each individual amplicon (respective reference sequence) during CRISPResso2 analyses. Data contained in files “Indel_histogram” and “CRISPResso_quantification_of_editing_frequency “ were used for preparation of Fig. 2 and Additional file 5: Figure S5. Raw data from amplicon sequencing is available on request.

### Long-read sequencing (PacBio)

Approximately 1 g of tissues derived from 2 week-old plants grown on 1/10 MS plates were used for DNA extraction. PacBio HiFi reads were filtered using BLASR [5] to remove the PacBio 2 kb sequence control. We employed a previously described approach for structural variant calling [26]. We performed *de novo* assemblies using Flye 2.9-b1768 [27] with an estimated genome size of 135 M and four polishing runs. We assessed the quality of the assembled contigs by (1) visualization with Bandage 0.8.1 [51], (2) calculation of the cumulative coverage and the N50 value as described [26], and (3) testing for the completeness of the assembly using BUSCO v5.2.2 in genome mode against the brassicales_odb10 database [34]. Next, contigs were used for scaffolding with Ragtag v2.1.0 [1] with default parameters and the TAIR10 Arabidopsis reference genome (https://ftp.ensemblgenomes.ebi.ac.uk/pub/plants/release-55/fasta/arabidopsis_thaliana/). To monitor the quality of the final assembly, we generated synteny plots of the final assembly using syri 1.6 [21] and plotsr 0.5.4 [20]. Structural variant calling was performed using SVIM-asm 1.0.2 [23] in haploid mode with–max_sv_size set to 2500 in order to exclude larger structural variants as a consequence of mis-assemblies. Putative variants containing undefined nucleotides (N’s) as well as the centromeric regions were excluded from the analysis. The resulting variants were annotated using snpEff 4.3t [7] using the TAIR10 genome annotation as reference feature file. For IGV visualization, reads or assemblies were aligned against the TAIR10 reference genome using minimap2 2.24-r1122 [30]. The complete pipeline is implemented in Python 3.8.5 and depends on seaborn, pandas, biopython as well as bash sub-processes and is deposited on Github (https://github.com/bubu227/deletion-of-genes-with-SpCas9/blob/main/pacbio_analysis_pipeline.py).

## Supplementary Information

**Additional file 1: Figure S1.** Expression of *zCas9i* in *N. benthamiana.*

**Additional file 2: Figure S2.** T_1_ deletion screening upon editing with two guide RNAs.

**Additional file 3: Figure S3.** T_1_ deletion screening upon editing with two guide RNAs.

**Additional file 4: Figure S4.** T_1_ deletion screening upon editing with six guide RNAs.

**Additional file 5: Figure S5.** Mutation (InDel) profiles in absence/presence of TREX2 at single target sites.

**Additional file 6: Figure S6.** PCR-genotyping of putative *wrky30* deletion lines in the T_2_ generation.

**Additional file 7: Figure S7.** Growth and development of *wrky30* mutant lines in comparison to Col-0.

**Additional file 8: Supplemental File S1.** Potential off-targets predicted by CRISPOR.

**Additional file 9: Supplemental File S2.** Plasmids, oligonucleotides, cloning details.

## Data Availability

All data is contained within the article or can be obtained through the authors. Plasmids pDGE1108 and pDGE1109 will be made available via Addgene. Pipeline for PacBio sequencing analysis is deposited on Github (https://github.com/bubu227/deletion-of-genes-with-SpCas9/blob/main/pacbio_analysis_pipeline.py).
