# Supplementary material for: Targeted gene deletion with SpCas9 and multiple guide RNAs in Arabidopsis thaliana: four are better than two
Source: Plant Methods. 2023 Mar 28;19:30. doi: 10.1186/s13007-023-01010-4 (PMC10053088; doi:10.1186/s13007-023-01010-4)
Supplement: Supplementary file 1 — Additional file 1: Figure S1. Expression of zCas9i in N. benthamiana. [file 13007_2023_1010_MOESM1_ESM.pdf]

Figure S1

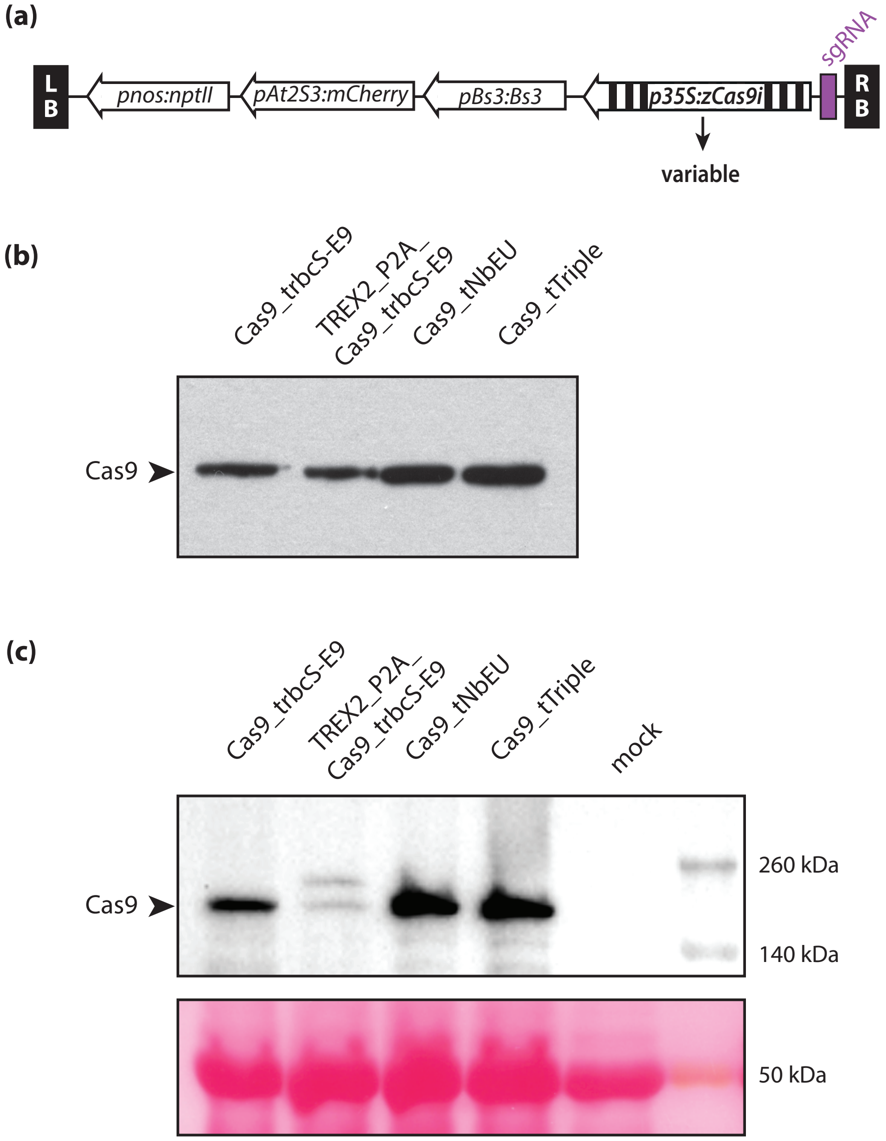

Figure S1: Expression of *zCas9i* in *N. benthamiana.*

a) Scheme of constructs used for expression in *N. benthamiana*. All constructs contained kanamycin-resistance as plant selectable marker, two cassettes designed for counter-selection (described in Stuttmann et al., 2021), an sgRNA expression unit and a cassette for zCas9i expression. Only the cassette for zCas9i expression was variable between constructs. zCas9i was expressed under 35S promoter control.

b) Immunodetection of Cas9. Agrobacterium strains for expression of Cas9 from the indicated cassettes were used for agroinfiltration at OD_{600}=0.4. Leaf discs were harvested three days post infiltration, ground in liquid nitrogen and boiled in Laemmli buffer for protein extraction. Proteins were separated on a 6 % PAA gel and transferred to a nitrocellulose membrane. Cas9 was detected using a rabbit monoclonal α-Cas9 antibody (Abcam EPR18991) and a HRP-coupled secondary antibody. Leaf discs originating from three independent replicates of the experiment were pooled for protein extraction. The experiment was conducted at the University of Halle. Similar results were obtained in two replicates.

c) similar to b), but the experiment was conducted at the Max Planck Institute for Plant Breeding research. Proteins were separated on a SERVA TG PRiME 10% gel and transferred to PVDF membranes. Cas9 was detected using a mouse monoclonal antibody (SAB4200701, Sigma-Aldrich). Ponceau staining is shown as loading control. Mock = buffer-infiltrated *N. benthamiana* tissues.
