# Supplementary material for: Targeted gene deletion with SpCas9 and multiple guide RNAs in Arabidopsis thaliana: four are better than two
Source: Plant Methods. 2023 Mar 28;19:30. doi: 10.1186/s13007-023-01010-4 (PMC10053088; doi:10.1186/s13007-023-01010-4)
Supplement: Supplementary file 3 — Additional file 3: Figure S3. T1 deletion screening upon editing with two guide RNAs. [file 13007_2023_1010_MOESM3_ESM.pdf]

Figure S3

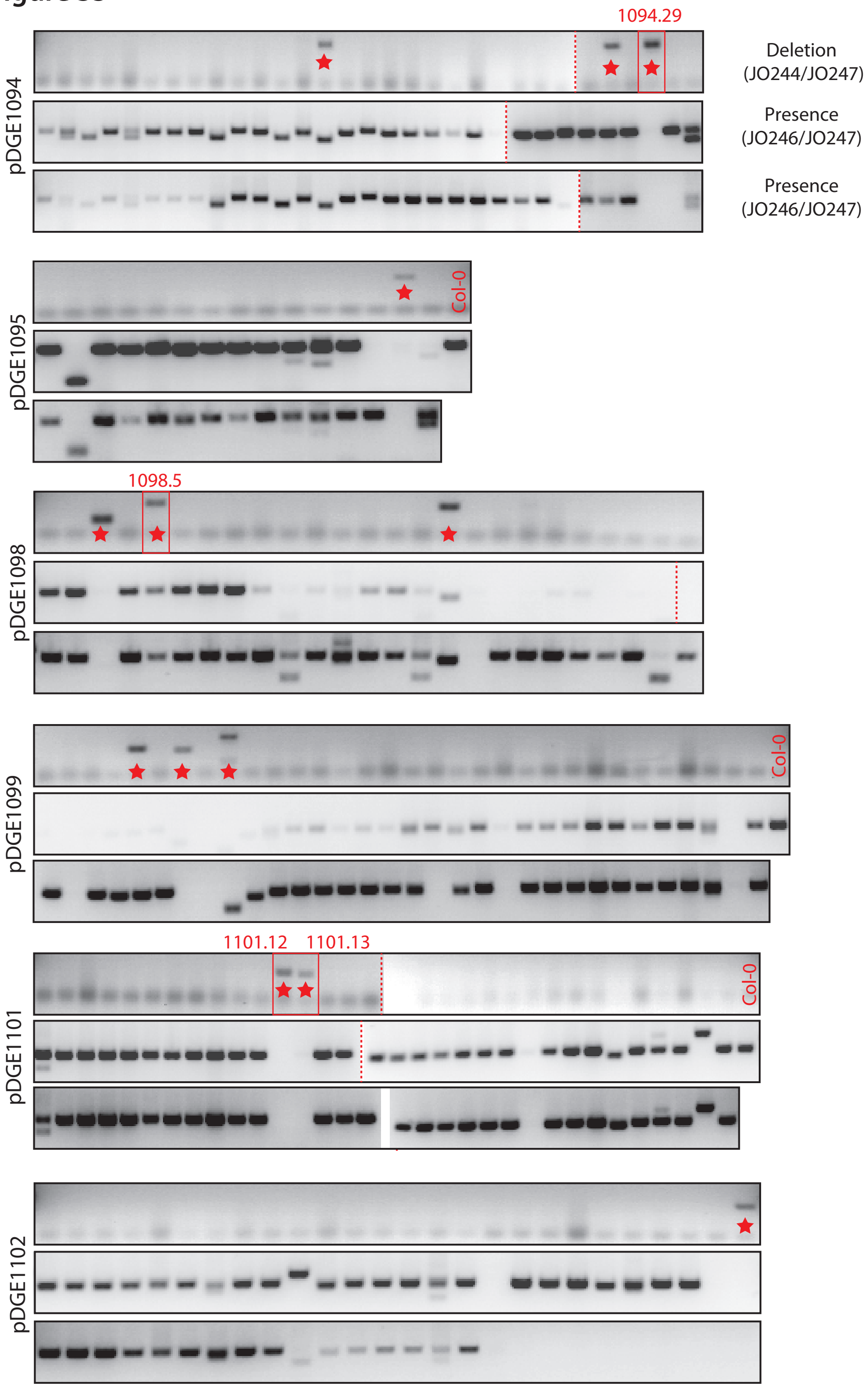

Figure S3: T_1 deletion screening upon editing with four guide RNAs.

Primary transformants (T_1) from transformation of indicated constructs were screened, by PCR, for presence of a large deletion encompassing the *WRKY30* locus (top PCR, oligonucleotides JO244/247). A second amplicon (oligonucleotides JO246/247) queries presence of the *WRKY30* locus, and serves as a control for DNA quality. PCR signals scored as presence of a deletion allele are marked with a star. Individuals for which neither PCR produced a signal were not counted. Grey boxes mask gel areas that were not considered, and dashed lines mark boundaries of spliced images. PCR signals corresponding to plants selected for further analysis in the T_2 generation are boxed and numbered (red).
