# Supplementary material for: Targeted gene deletion with SpCas9 and multiple guide RNAs in Arabidopsis thaliana: four are better than two
Source: Plant Methods. 2023 Mar 28;19:30. doi: 10.1186/s13007-023-01010-4 (PMC10053088; doi:10.1186/s13007-023-01010-4)
Supplement: Supplementary file 4 — Additional file 4: Figure S4. T1 deletion screening upon editing with six guide RNAs. [file 13007_2023_1010_MOESM4_ESM.pdf]

Figure S4

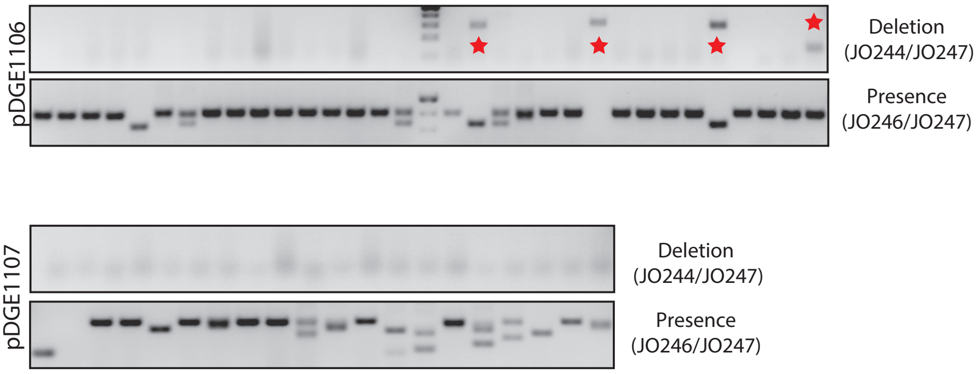

Figure S4: T_1 deletion screening upon editing with six guide RNAs.

Primary transformants (T_1) from transformation of indicated constructs were screened, by PCR, for presence of a large deletion encompassing the *WRKY30* locus (top PCR, oligonucleotides JO244/247). A second amplicon (oligonucleotides JO246/247) queries presence of the *WRKY30* locus, and serves as a control for DNA quality. PCR signals scored as presence of a deletion allele are marked with a star. Individuals for which neither PCR produced a signal were not counted. Grey boxes mask gel areas that were not considered, and dashed lines mark boundaries of spliced images.
