# Supplementary material for: Targeted gene deletion with SpCas9 and multiple guide RNAs in Arabidopsis thaliana: four are better than two
Source: Plant Methods. 2023 Mar 28;19:30. doi: 10.1186/s13007-023-01010-4 (PMC10053088; doi:10.1186/s13007-023-01010-4)
Supplement: Supplementary file 5 — Additional file 5: Figure S5. Mutation (InDel) profiles in absence/presence of TREX2 at single target sites. [file 13007_2023_1010_MOESM5_ESM.pdf]

Figure S5

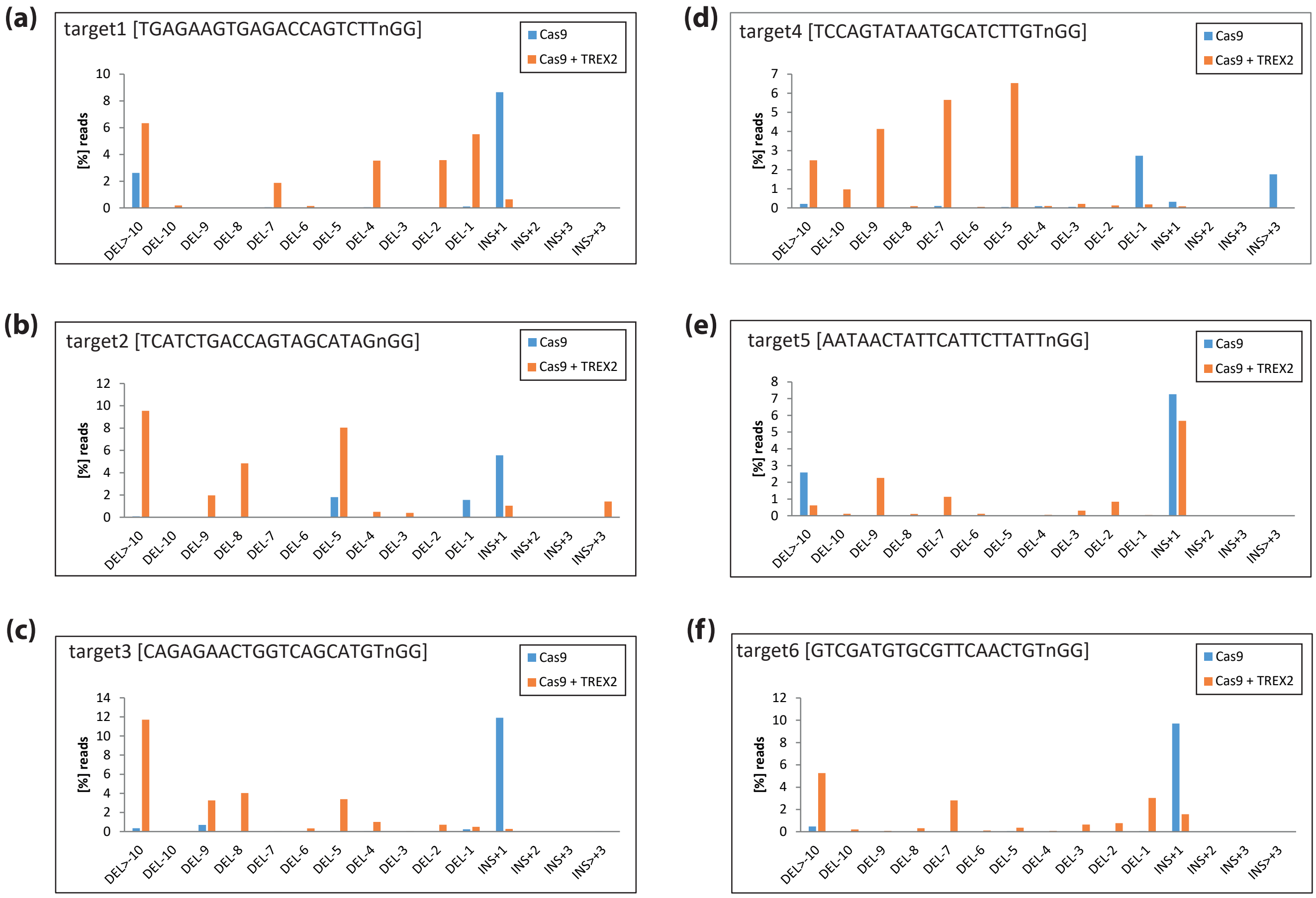

Figure S5: Mutation (InDel) profiles in absence/presence of TREX2 at single target sites.

Panels a)-f) show InDel profiles as determined by amplicon sequencing and CRISPresso analysis for individual target sites. For example, graphs a) and b) represent data obtained using DNA from T_1 transformants from pDGE1081 and pDGE1082.
