# Supplementary material for: Targeted gene deletion with SpCas9 and multiple guide RNAs in Arabidopsis thaliana: four are better than two
Source: Plant Methods. 2023 Mar 28;19:30. doi: 10.1186/s13007-023-01010-4 (PMC10053088; doi:10.1186/s13007-023-01010-4)
Supplement: Supplementary file 6 — Additional file 6: Figure S6. PCR-genotyping of putative wrky30 deletion lines in the T2 generation. [file 13007_2023_1010_MOESM6_ESM.pdf]

Figure S6

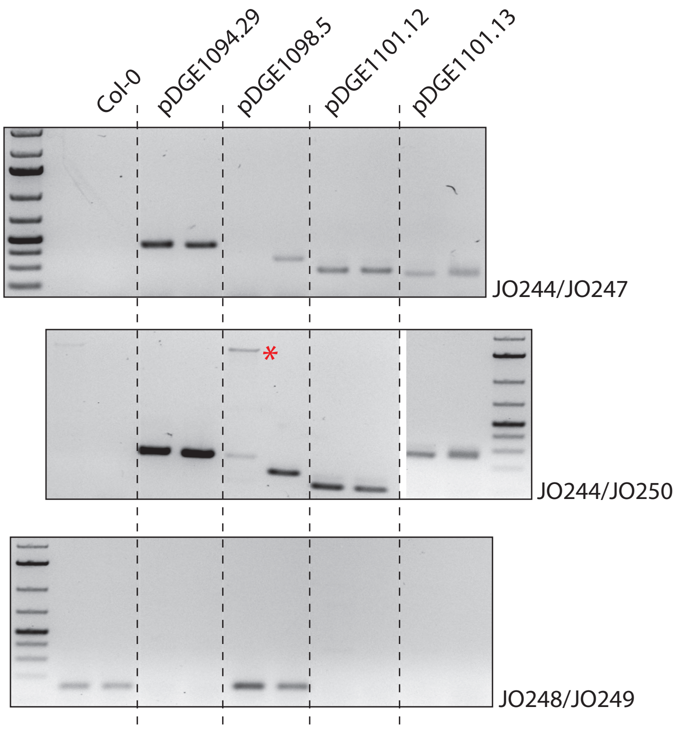

Figure S6: PCR-genotyping of putative *wrky30* deletion lines in the T_2 generation.

Non-transgenic seeds were selected from indicated T_2 populations (see Figure S4 for genotyping in the T_1 generation) using the FAST marker. Four plants were pooled for DNA extraction; two pools per population were genotyped by PCR for presence/absence (deletion) of the *WRKY30* locus. Amplicons JO244/247 and JO244/250 both query deletion of *WRKY30* (see Figure 1 for primer binding sites). A product corresponding to the length of the wild-type *WRKY30* locus was amplified from one pool DNA derived from 1098.5 (marked with an asterisk). Amplicon JO248/249 queries presence of the *WRKY30* locus. PCR results confirm bi-allic deletions at the *WRKY30* locus (Figure S4) for populations 1094.29, 1101.12 and 1101.13.
