# Supplementary material for: Targeted gene deletion with SpCas9 and multiple guide RNAs in Arabidopsis thaliana: four are better than two
Source: Plant Methods. 2023 Mar 28;19:30. doi: 10.1186/s13007-023-01010-4 (PMC10053088; doi:10.1186/s13007-023-01010-4)
Supplement: Supplementary file 7 — Additional file 7: Figure S7. Growth and development of wrky30 mutant lines in comparison to Col-0. [file 13007_2023_1010_MOESM7_ESM.pdf]

Figure S7

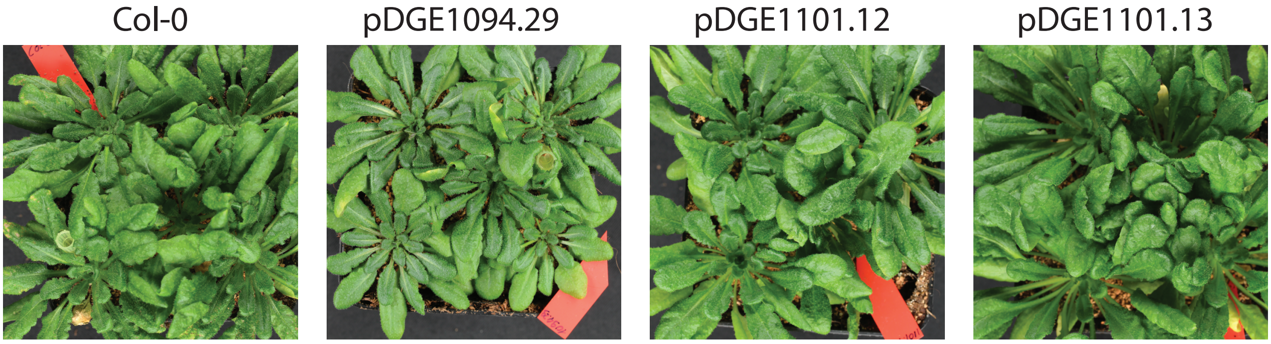

Figure S7: Growth and development of *wrky30* mutant lines in comparison to Col-0.

Transgene-free, bi-allelic *wrky30* mutant plants from the indicated populations were grown side-by-side with control plants (Col-0) in short day. Macroscopic growth phenotypes were documented after five weeks. No differences were observed between *wrky30* plants and the corresponding wild type.
