# Supplementary material for: Targeted gene deletion with SpCas9 and multiple guide RNAs in Arabidopsis thaliana: four are better than two
Source: Plant Methods. 2023 Mar 28;19:30. doi: 10.1186/s13007-023-01010-4 (PMC10053088; doi:10.1186/s13007-023-01010-4)
Supplement: Supplementary file 9 — Additional file 9: Supplemental File S2. Plasmids, oligonucleotides, cloning details. [file 13007_2023_1010_MOESM9_ESM.pdf]

Supplemental File S2: Plasmids, oligonucleotides, cloning details

Previously published plasmids:

name	Description	Reference
pDGE331	M1E sgRNA shuttle	(Stuttmann *et al.*, 2021)
pDGE332	M1 sgRNA shuttle	(Stuttmann *et al.*, 2021)
pDGE333	M2 sgRNA shuttle	(Stuttmann *et al.*, 2021)
pDGE334	M2E sgRNA shuttle	(Stuttmann *et al.*, 2021)
pDGE335	M3 sgRNA shuttle	(Stuttmann *et al.*, 2021)
pDGE336	M4 sgRNA shuttle	(Stuttmann *et al.*, 2021)
pDGE337	M4E sgRNA shuttle	(Stuttmann *et al.*, 2021)
pDGE495	M5 sgRNA shuttle	(Stuttmann *et al.*, 2021)
pDGE497	M6E sgRNA shuttle	(Stuttmann *et al.*, 2021)
pCK70	zCas9i(2xNLS) (pAGM47523) [CDS1]	(Stuttmann *et al.*, 2021)<br>(Grützner *et al.*, 2021)
pCK73	p2x35S:zCas9i-tRbcSE9 [1-2f]	(Stuttmann *et al.*, 2021)
pCK75	pBs3:Bs3-tocs [1-3f]	(Stuttmann *et al.*, 2021)
pCK76	pnos:nptII-tnos [1-5f]	(Stuttmann *et al.*, 2021)
pTEI44	pRPS5a [Pro+5U(f)]	(Gantner *et al.*, 2018)
pJOG292	Acceptor for pDGE recipient assembly	(Ordon *et al.*, 2017)
pJOG294	ccdB cassette for pDGE recipient assembly	(Ordon *et al.*, 2017)
pJOG416	trbcS-E9 [3U+ter]	(Gantner *et al.*, 2018)
pJOG603	pRPS5a [Pro+5U]	(Gantner *et al.*, 2018)
pJOG640	p35S [Pro+5U(f)]	(Gantner *et al.*, 2018)
pJOG648	pAt2S3 [Pro+5U]	(Gantner *et al.*, 2018)
pJOG990	NbEU terminator [3U+Ter]	(Stuttmann *et al.*, 2021)<br>(Diamos and Mason, 2018)
pJOG1008	t35S::tNbAct::Rb7MAR terminator (ttriple) [3U+Ter]	(Diamos and Mason, 2018,<br>Stuttmann *et al.*, 2021)
pICH47761	Level 1 acceptor	(Engler *et al.*, 2014)
pICH47742	Level 1 acceptor	(Engler *et al.*, 2014)
pICH47751	Level 1 acceptor	(Engler *et al.*, 2014)
pICH41800	End-linker	(Engler *et al.*, 2014)
pICH41766	End-linker	(Engler *et al.*, 2014)
pICHSL80007	mCherry [CDS1]	(Engler *et al.*, 2014)
pICH72400	tug7 [3U+ter]	(Engler *et al.*, 2014)
pICH51288	2x35S [Pro+5U]	(Engler *et al.*, 2014)
pICSL70008	FAST [gene]	(Engler *et al.*, 2014)

Level 0 modules (cloned as described in Engler at al., 2014):

name	recipient	Description	oligonucleotides
pJOG983	pGAM1276	TREX2-P2A as NT1	JS1684/1685 on plasmid Addgene #91026 (Cermak et al., 2017)

Level 1 modules (assembled as described in Engler et al., 2014):

name	recipient	Inserts	Description
pJOG685	pICH47761	pJOG648, pICHSL80007, pICH72400	pAtS2S3:mCherry-tug7 [1-4f]
pJOG991	pICH47742	pJOG640, pJOG983, pJOG416, pCK70	p35S:TREX-zCas9i-trbcS [1-2f]
pJOG1030	pICH47742	pICH51288, pCK70, pJOG990	p35S:zCas9i-tNbEU [1-2f]
pJOG1031	pICH47742	pICH51288, pCK70, pJOG1008	p35S:zCas9i-ttriple [1-2f]
pJOG304	pICH47751	pICSL70008	FAST [1-3f]
pCK226	pICH47742	pJOG603, pCK70, pJOG1008	pRPS5a:zCas9i_ttriple [1-2f]
pCK227	pICH47742	pTEI44, pJOG983, pCK70, pJOG1008	pRPS5a:TREX2-zCas9i_ttriple [1-2f]

pDGE – recipients (assembled as described for Level 2 constructs in Engler et al., 2014):

name	recipient	inserts	description
pDGE1108	pJOG292	pJOG304, pCK226, pJOG294, pICH41766	FAST_ pRPS5a:Cas9(ttriple)_ccdB
pDGE1109	pJOG292	pJOG304, pCK227, pJOG294, pICH41766	FAST_ pRPS5a:TREX-Cas9(ttriple)_ccdB
pDGE311	pJOG292	pCK73, pCK75, pCK76, pJOG685, pJOG294, pICH41800	nptII-Bs3-Cherry-35S:Cas9_rbcS-ccdB
pDGE345	pDGE311	pDGE331 (empty M1E module)	nptII-Bs3-Cherry-35S:Cas9_rbcS-M1E
pDGE355	pJOG292	pJOG294, pJOG685, pJOG991, pCK75, pCK76, pICH41800	nptII-Bs3-Cherry-35S:TREX2-Cas9_rbcS-ccdB
pDGE399	pJOG292	pJOG1030, pCK75, pCK76, pJOG685, pJOG294, pICH41800	nptII-Bs3-Cherry-35S:Cas9_NbEU-ccdB
pDGE400	pJOG292	pJOG1031, pCK75, pCK76, pJOG685, pJOG294, pICH41800	nptII-Bs3-Cherry-35S:Cas9_ttriple-ccdB

pDGE - sgRNA shuttle vectors (constructed as described in Stuttmann et al., 2021):

name	recipient	Oligonucleotides	Module type
pDGE390	pDGE331	JS809/810	M1E
pDGE1079	pDGE332	JS2642/2643	M1
pDGE1080	pDGE334	JS2648/2649	M2E
pDGE1083	pDGE332	JS2644/2645	M1
pDGE1084	pDGE334	JS2646/2647	M2E
pDGE1087	pDGE332	JS2640/2641	M1
pDGE1088	pDGE334	JS2650/2651	M2E
pDGE1091	pDGE333	JS2644/2645	M2
pDGE1092	pDGE335	JS2648/2649	M3
pDGE1093	pDGE337	JS2646/2647	M4E
pDGE1096	pDGE333	JS2640/2641	M2
pDGE1097	pDGE337	JS2650/2651	M4E
pDGE1100	pDGE335	JS2650/2651	M3
pDGE1103	pDGE336	JS2640/2641	M4
pDGE1104	pDGE495	JS2646/2647	M5
pDGE1105	pDGE497	JS2650/2651	M6E

pDGE – editing constructs (assembled as described in Stuttmann et al., 2021):

name	recipient	inserts	description
pDGE375	pDGE345	Oligonucleotides JS809/810	nptII-Bs3-Cherry-35S:Cas9_rbcS-sgRNA
pDGE404	pDGE355	pDGE390 (sgRNA vs. NbEDS1)	nptII-Bs3-Cherry-35S:TREX2-Cas9_rbcS-sgRNA
pDGE405	pDGE399	pDGE390 (sgRNA vs. NbEDS1)	nptII-Bs3-Cherry-35S:Cas9_NbEU-sgRNA
pDGE406	pDGE400	pDGE390 (sgRNA vs. NbEDS1)	nptII-Bs3-Cherry-35S:Cas9_ttriple-sgRNA
pDGE1081	pDGE1108	pDGE1079, 1080	WRKY editing 2 sgRNAs
pDGE1082	pDGE1109	pDGE1079, 1080	WRKY editing 2 sgRNAs TREX
pDGE1085	pDGE1108	pDGE1083, 1084	WRKY editing 2 sgRNAs
pDGE1086	pDGE1109	pDGE1083, 1084	WRKY editing 2 sgRNAs TREX
pDGE1089	pDGE1108	pDGE1087, 1088	WRKY editing 2 sgRNAs
pDGE1090	pDGE1109	pDGE1087, 1088	WRKY editing 2 sgRNAs TREX
pDGE1094	pDGE1108	pDGE1079, 1091,1092,1093	WRKY editing 4 sgRNAs
pDGE1095	pDGE1109	pDGE1079, 1091,1092,1093	WRKY editing 4 sgRNAs TREX
pDGE1098	pDGE1108	pDGE1079,1096,1092, 1097	WRKY editing 4 sgRNAs
pDGE1099	pDGE1109	pDGE1079,1096,1092, 1097	WRKY editing 4 sgRNAs TREX
pDGE1101	pDGE1108	pDGE1083, 1096, 1100,1093	WRKY editing 4 sgRNAs
pDGE1102	pDGE1109	pDGE1083, 1096, 1100,1093	WRKY editing 4 sgRNAs TREX
pDGE1106	pDGE1108	pDGE1079,1091,1092, 1103,1104,1105	WRKY editing 6 sgRNAs
pDGE1107	pDGE1109	pDGE1079,1091,1092, 1103,1104,1105	WRKY editing 6 sgRNAs TREX

Oligonucleotides used for cloning of Level 0 modules:

name	sequence
JS1684	ttgaagacatCCATGTCTGAGCCACCTCGGGCTGAaACCTTTG
JS1685	ttgaagacatCATtGGTCCAGGATTTTCTTCAAC

Oligonucleotides for sgRNA construction:

name	sequence
JS809	ATTGTATGCTGCATGTAATCTGAA
JS810	AAACTTCAGATTACATGCAGCATA
JS2640	attgAATAACTATTCATTCTTATT
JS2641	aaacAATAAGAATGAATAGTTATT
JS2642	attgTGAGAAGTGAGACCAGTCTT
JS2643	aaacAAGACTGGTCTCACTTCTCA
JS2644	attgCAGAGAACTGGTCAGCATGT
JS2645	aaacACATGCTGACCAGTTCTCTG
JS2646	attgTCCAGTATAATGCATCTTGT
JS2647	aaacACAAGATGCATTATACTGGA
JS2648	attgTCATCTGACCAGTAGCATAG
JS2649	aaacCTATGCTACTGGTCAGATGA
JS2650	attGTCGATGTGCGTTCAACTGT
JS2651	aaacACAGTTGAACGCACATCGA

Oligonucleotides used for genotyping, sequencing:

name	Sequence [purpose]	
JS1753	GCGATCAGATTCTCAAGCCG	[zCas9i presence/absence]
JS1754	TTTTGCAGGTTGACGACTCG	[zCas9i presence/absence]
JO244	TTGTCTCTTGGAATTTCTAAACTCAA	[WRKY30 genotyping]
JO245	TTTGACTGAAGAACGAAGAAAGCT	[WRKY30 genotyping]
JO246	TGCAAATTTGAGTCTTCTTTTAGCT	[WRKY30 genotyping]
JO247	TCTGTGGTAGAGAAATTAAAGAGGT	[WRKY30 genotyping]
JO248	CCACTCTTTGAACGTAATGGAGAA	[WRKY30 genotyping]
JO249	TTTGGCTAAATGTTCACGTGTTTC	[WRKY30 genotyping]
JO250	AGAAAAGTTTATCTGTCTGTGGTAGA	[WRKY30 genotyping]
JS1132	AACGCTCTTTTCTCTTAGGT	[sgRNA array sequencing]
JS2302	GTAATAGCAATGACCAGTGC	[sgRNA array sequencing]

References

Diamos, A.G. and Mason, H.S. (2018) Chimeric 3' flanking regions strongly enhance gene expression in plants. *Plant Biotechnol J*.

Engler, C., Youles, M., Gruetzner, R., Ehnert, T.M., Werner, S., Jones, J.D., Patron, N.J. and Marillonnet, S. (2014) A Golden Gate Modular Cloning Toolbox for Plants. *ACS synthetic biology*.

Gantner, J., Ordon, J., Ilse, T., Kretschmer, C., Gruetzner, R., Lofke, C., Dagdas, Y., Burstenbinder, K., Marillonnet, S. and Stuttmann, J. (2018) Peripheral infrastructure vectors and an extended set of plant parts for the Modular Cloning system. *PLoS ONE*, **13**, e0197185.

Grützner, R., Martin, P., Horn, C., Mortensen, S., Cram, E.J., Lee-Parsons, C.W.T., Stuttmann, J. and Marillonnet, S. (2021) High-efficiency genome editing in plants mediated by a Cas9 gene containing multiple introns. *Plant Communications*, **2**, 100135.

Ordon, J., Gantner, J., Kemna, J., Schwalgun, L., Reschke, M., Streubel, J., Boch, J. and Stuttmann, J. (2017) Generation of chromosomal deletions in dicotyledonous plants employing a user-friendly genome editing toolkit. *Plant J*, **89**, 155-168.

Stuttmann, J., Barthel, K., Martin, P., Ordon, J., Erickson, J.L., Herr, R., Ferik, F., Kretschmer, C., Berner, T., Keilwagen, J., Marillonnet, S. and Bonas, U. (2021) Highly efficient multiplex editing: one-shot generation of 8x Nicotiana benthamiana and 12x Arabidopsis mutants. *Plant J*, **106**, 8-22.
